# Ampicillin Silver Nanoformulations against Multidrug resistant bacteria

**DOI:** 10.1038/s41598-019-43309-0

**Published:** 2019-05-02

**Authors:** Nafeesa Khatoon, Hammad Alam, Afreen Khan, Khalid Raza, Meryam Sardar

**Affiliations:** 1 Department of Biosciences, Jamia Millia Islamia, New Delhi, 110025 India; 2 Department of Computer Science, Jamia Millia Islamia, New Delhi, 110025 India

**Keywords:** Chemical biology, Biotechnology

## Abstract

The present study reported a single step synthesis of silver nanoparticles using ampicillin (Amp-AgNps), a second-generation β lactam antibiotic to get nanoformulation having dual properties that of antibiotic and silver. The Amp-AgNps was characterized by UV-VIS spectroscopy, TEM, XRD, FTIR and TGA. FTIR and TGA results suggested that amine group of Ampicllin reduce the metalic silver into nano form. These results were further validated by computational molecular dynamics simulation. The antibacterial potential of Amp-AgNps was investigated against sensitive and drug resistant bacteria. MIC of Amp-AgNps against 6 different bacterial strains were in the range of 3–28 µg/ml which is much lower than the MIC of ampicillin (12–720 µg/ml) and chemically synthesized silver nanoparticles (280–640 µg/ml). The repeated exposure to drugs may lead to development of resistance mechanism in bacteria against that drug, so the efficacy of Amp-AgNps after repeated exposure to bacterial strains were also studied. The results indicate that bacterial strains do not show any resistance to these Amp-AgNps even after exposure up to 15 successive cycles. The biocompatibility of these Amp-AgNps was checked against cell lines by using Keratinocytes cell lines (HaCaT).

## Introduction

Nanotechnology is the branch of science which deals in tuning and modifying the organic or inorganic materials into desired and unique nanomaterials. These materials are gaining importance in medicine^1,2^, pharmaceuticals^3^, cosmetics^4^, food and beverages^5^, paper and pulp and many more industries^6^. Medicate resistance is a noteworthy issue worldwide and the boundless utilization of expensive range of anti-infection agents has delivered resistance against pathogenic microbes. Among medication, numerous extensive variety of anti-microbial agents are economically accessible against Gram-positive and Gram negative microscopic organisms but the wide utilization of these agents has made microorganisms create resistance against them. There are several mechanism by which bacteria develop resistance against the antibiotics example some microbes express enzymes that modify and degrade the antibiotics, modification of cellular component and formation of efflux pumps are another mode of resistance^2^. The endeavors have been made to defeat the resistance system by different methodologies, for example, to adjust the structure of β-lactam moiety of anti-infection agents and to integrate new anti-microbials of high productivity despite the fact that these techniques are tedious and are frequently not monetarily possible. To overcome, there is a requirement for the improvement of new and compelling drugs and restorative methodologies that provide assistance in fighting medication resistance.

Studies revealed that the metal nanoparticles can be used as effective antimicrobial agents^7^. Among metals, silver nanoparticles are widely used in catalysis^8^, bioremediation^9^, bioimaging^10^, sensors^11^ and in antimicrobial therapies^12^. Though, the researchers have widely explored the antibacterial efficacy of silver nanoparticle but their mode of action is still not clear^13,14^. They show antimicrobial activity against different types of microorganisms^12^. Moreover, silver nanoparticles modified with different stabilizers such as polymers, carbohydrates, amino acids and antibiotics generally showed an increased antibacterial activity^15–17^. Brown *et al*. 2012 reported the adsorbtion of ampicillin on citrate coated chemically synthesized silver and gold nanoparticles^17^. Their study suggested that the antibiotic attaches to silver/gold nanoparticles via thioether moiety^17^. Rogowska *et al*. 2017, compared the binding/adsorption of ampicillin to chemically synthesized nanoparticles (20 nm) coated with sodium citrate and biologically synthesized nanoparticles (by Actinomycetes strain CGG 11n, average size 17 nm). They observed that adsorption of ampicillin on citrate capped silver nanoparticles is less stable compared to biologically synthesized may be due to disequilibrium in binding of silver to the negatively charged citrate^18^. The ampicillin adsorbed to biologically synthesized nanoparticles, is more stable as these nanoparticles are coated with an organic layer which acts as a stabilizer^18^. Moreover, physical adsorption may cause leaching of the drug from the nanoparticles. Oliveira *et al*. 2017 reported an effective antimicrobial nanoantibiotic by covalently attaching ampicillin to core-shell Ag@SiO_2_. AgNPs were formed by the chemical reduction of silver nitrate then a silica shell was produced by hydrolysis and condensation of tetraethyl orthosilicate, the core-shell Ag@SiO_2_ nanoparticles then coated with 3-aminopropyl triethoxysilane. Finally, the amino-functionalized nanoparticles were reacted with ampicillin through an acid-base coupling reaction^19^. Thus, there is a need to develop new and efficient protocols to modify silver nanoparticles with conventional drugs. Rai *et al*. 2010 reported the synthesis of gold nanoparticles using second generation antibiotic cefaclor and their antimicrobial activity against gram positive and gram negative bacteria^20^. There are very few reports on the synthesis of gold nanoparticles using antibiotics but so far no reports are available for silver nanoparticle synthesis using antibiotics. Therefore, in the present work silver nanoparticles were synthesized in a one step reaction using ampicillin as a sole reducing and capping agent, so that synthesized silver nanoparticles posses dual properties that of ampicillin and silver nano.

## Results

**Figure 1:**
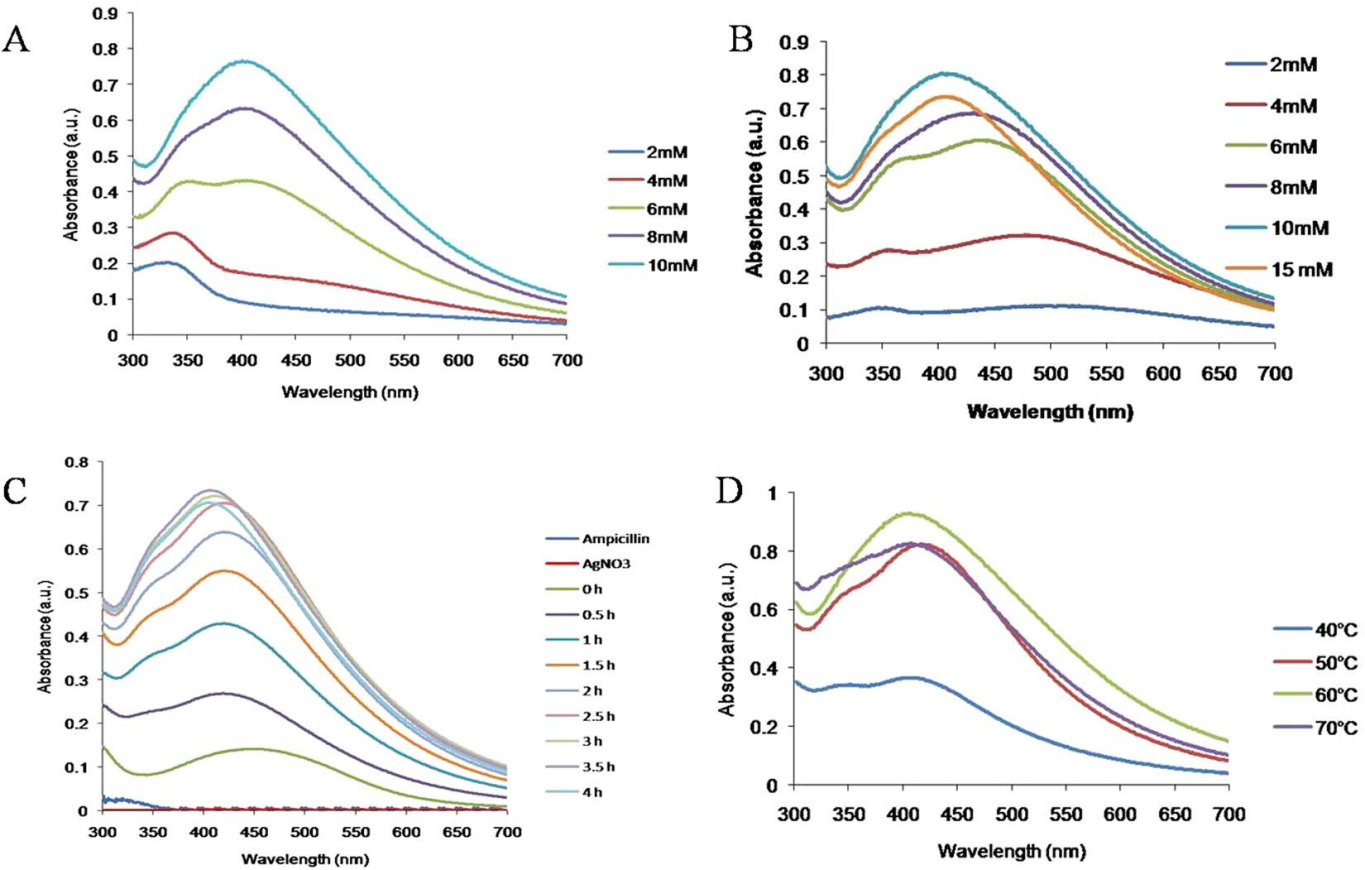
UV-Vis spectra recorded as a function of concentration of silver nitrate (**A**), Ampicillin (**B**), time (**C**) and temperature (**D**) for Amp-AgNps.

**Figure 2:**
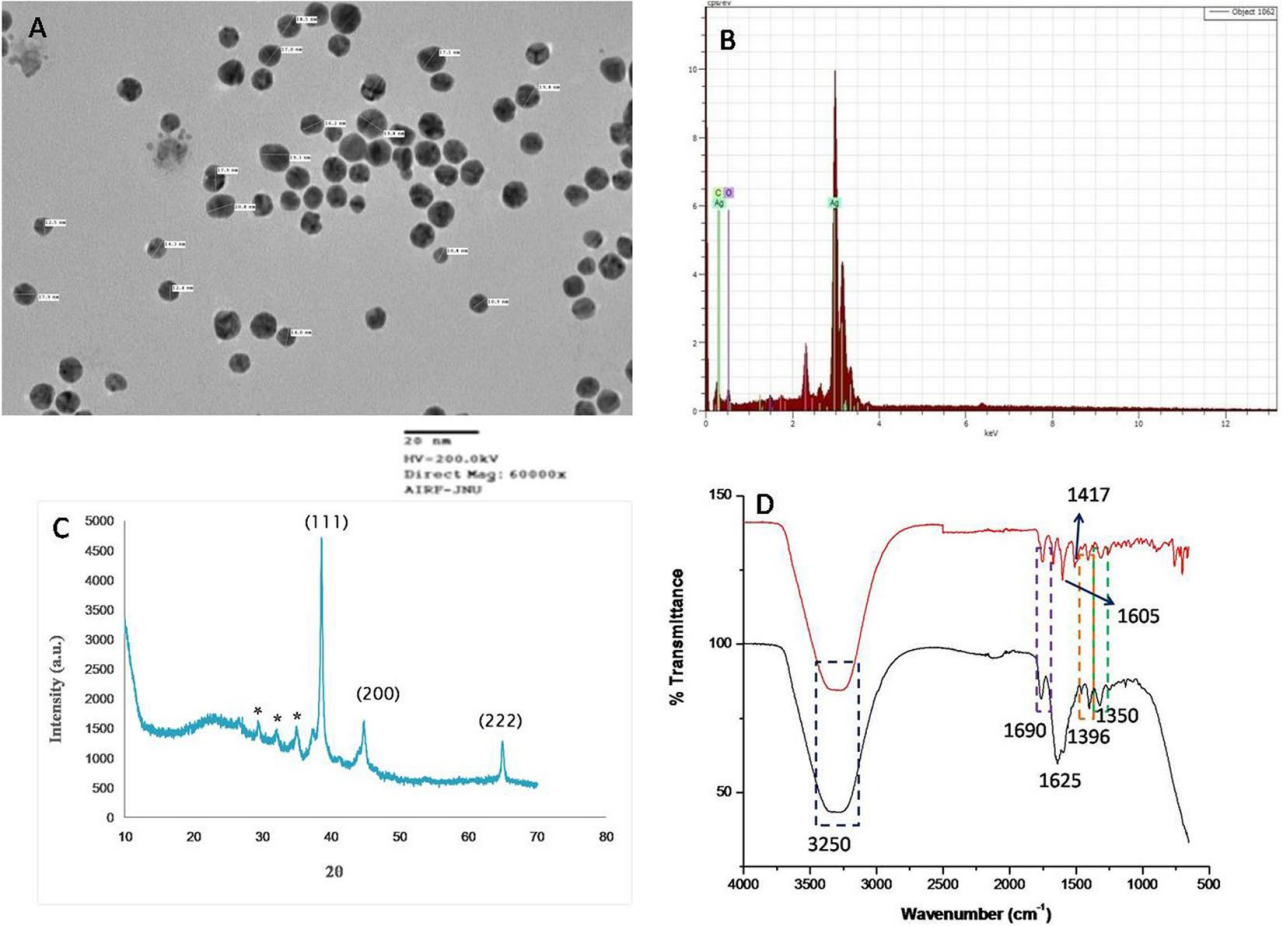
TEM micrograph (**A**), EDX (**B**), XRD pattern (**C**) and FTIR (**D**) of Ampicillin (red) and Amp-AgNps (black).

**Figure 3:**
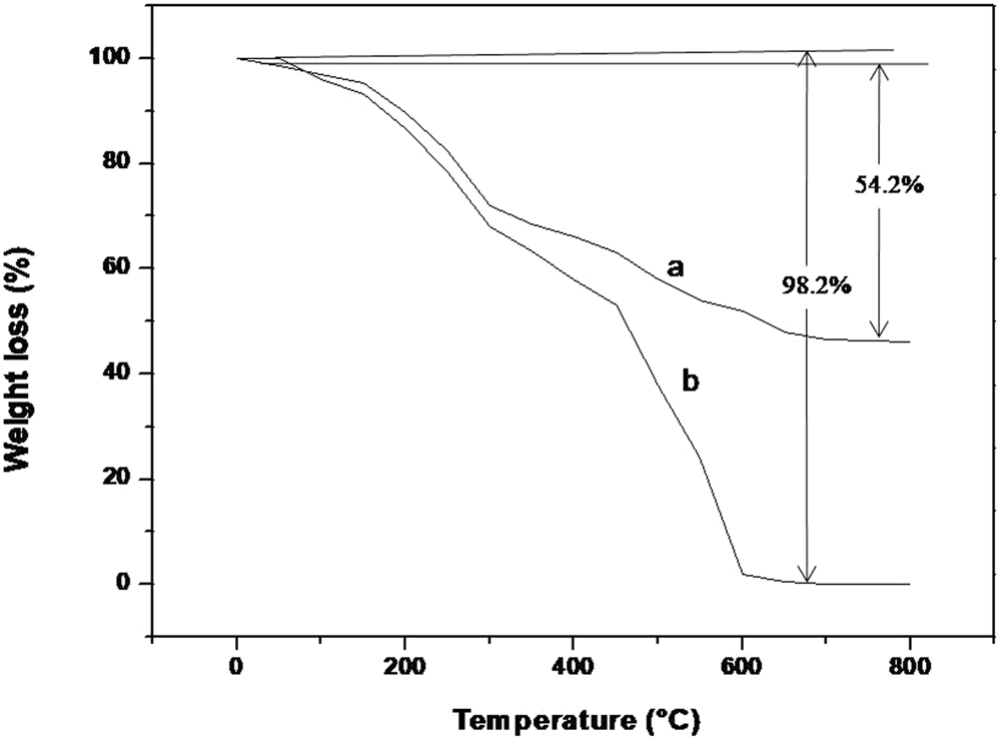
TGA analysis of Amp-AgNps (curve a) and Ampicillin (curve b).

**Figure 4:**
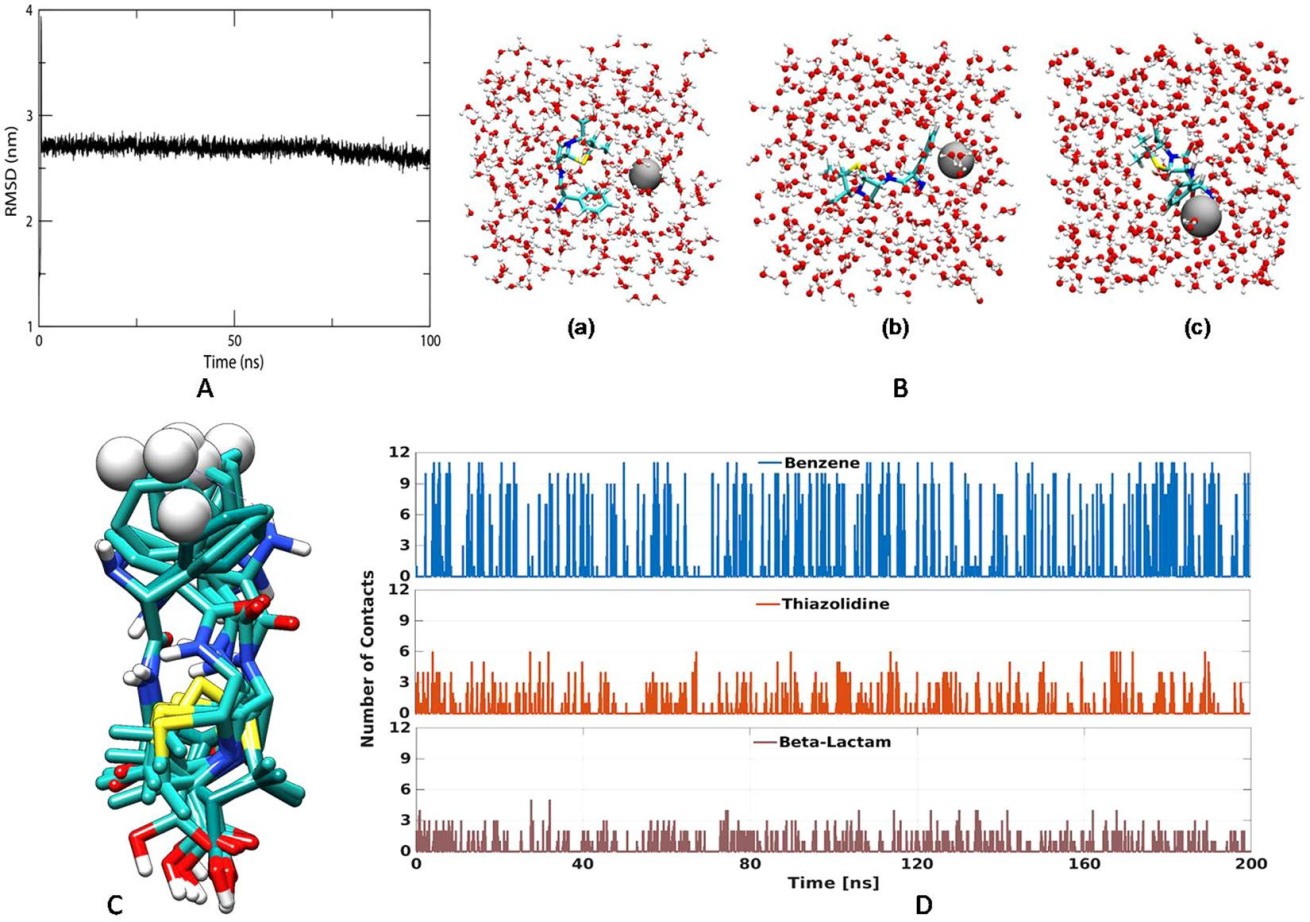
(**A**) RMSD plot for molecular dynamic simulation of Amp-AgNps. (**B**) Initial structure of ampicillin and silver ion in water (a), structure of Amp-AgNps at 100 ns (b) and 200 ns (c). (**C**) Interaction of Silver with benzene ring of Ampicillin at different time frame. (**D**) Contacts of Silver with various groups of Ampicillin with respect to time.

Ampicillin is a semi-synthetic derivative of penicillin, a β lactam antibiotic that functions as an orally active broad-spectrum drug. It has been used extensively to treat bacterial infections since 1961. The extensive use of antibiotics results in the development of bacterial resistance against them^2^. The number of papers have reported that silver nanoparticles are effective against drug resistance bacteria but Panaeck *et al*. 2018 reported that the repeated use of chemically synthesized silver nanoparticles led to the emergence of bacterial resistance against gram negative bacteria^21^. This resistance is due to the production of flagellum protein flagellin, which causes aggregation of nanoparticles, thus new strategies should be developed to enhance the bactericidial properties of silver nanoparticles. Mohler *et al*. 2017 reported that conjugation of metal ions to β-lactam antibiotics can enhance their antimicrobial properties^22^. Thus in the present study synthesis of silver nanoparticles was carried out by the reduction of silver ions using ampicillin. The concentration of metal salt, reducing agents, temperature and time plays important role in synthesis, thus all these parameters has been optimized. The formation of Amp-AgNps was monitored by varying the concentrations of silver nitrate and ampicillin from 2 mM to 15 mM, the results show that as the concentration of silver nitrate/ampicillin increases the absorption intensity increases (Fig. 1A,B) these results were in agreement with earlier published results^20^. The maximum synthesis was achieved at a concentration of 10 mM in both cases. The λ_max_ of brown color solution was obtained at 406 nm after 3.5 hr that is characteristic of the surface plasmon resonance of silver nanoparticles (Fig. 1C). The controls of ampicillin and silver nitrate separately did not show any peak in the visible region indicating that ampicillin is responsible for the synthesis of silver nanoparticles. Figure 1D corresponds the UV-Vis spectrum of Amp-AgNps at a temperature of range 40–70 °C. The spectrum shows that as the temperature increases from 40 °C to 60 °C, the intensity of synthesis increases and maximum was achieved at 60 °C, the SPR band is also shifted to lower wavelength from 40 °C at 420 to 60 °C at 406 nm. The blue shift in SPR band is characteristic of smaller size silver nanoparticle synthesis. Beyond 60 °C the SPR band is constant only intensity of formation has been reduced. This may be due to hydrolysis and decarboxylation of ampicillin at high temperature^23^. The stability of these Amp-AgNPs was studied by keeping them at room temperature for three month in solution phase. The UV-Vis spectra were recorded and no change in SPR was observed indicating that Amp-AgNPs are highly stable. The DLS studies were carried out to determine the hydrodynamic radius of Amp-AgNps which reveals that silver nanoparticles have average diameter of 44.12 ± 2.89 nm. The particle size distribution was determined by calculating Polydispersity index (PDI) and PDI was found to be 0.32. Further zeta potentials was determined to check the stability of colloidal suspensions and was found to be +33.42 mV, which indicate a long-term stability of Amp-AgNps in suspension phase. To know about the size and morphology, TEM analysis of Amp-AgNps were performed. TEM micrographs shows that the Amp-AgNps are spherical of diameter 9–20 nm (Fig. 2A). The elemental analysis was performed by EDX which confirms the formation of Amp-AgNps (Fig. 2B). XRD data shows the peaks of cubic phase at 111, 200, 220 (Fig. 2C) which confirms the crystalline nature of Amp-AgNps. XRD results correspond to JCPD file no. 03–0921. The FTIR spectrum of only ampicillin shows the bands at 3250, 1690, 1605, 1417, 1396 and 1350 where 3250 correspond to -OH, 1690 is C=O stretching vibrations of four membered ring lactams^20^, 1605 primary amine and peak at 1417, 1396 and 1350 were contributed to characteristic β lactam ring vibrations (Fig. 2D)^20^. The peak at 1625 cm^−1^ for Amp-AgNps is due to amine group, that has been shifted to a higher wavenumber in comparison to the primary amine of pure ampicillin (1605 cm^−1^), suggested the role of primary amine group of ampicillin in reduction of Amp-AgNps. The β-lactam ring bands and other bands do not show any shift in Amp-AgNps as compared to pure ampicillin. Hence it can be inferred that while formation of Amp-AgNps, β-lactam ring of ampicillin preserves its integrity. Similar, result has been reported by Rai *et al*., 2010 on the formation of gold nanoparticles using cefaclor antibiotic as a reducing agent. The primary amine group of cefaclor acted as both the reducing and capping agent for the synthesis of gold nanoparticles leaving the β -lactam ring unaffected^20^. The ampicillin content of Amp-AgNps was evaluated by thermogravimetric analysis (TGA) in order to obtain quantitative amount of ampicillin (Fig. 3). The weight loss curve of ampicillin and Amp-AgNps reveals three different weight losses. Initial 2.1% to 4.3% weight loss in the temperature region 30–200 °C which may be due to physical adsorption of ampicillin on the surface of Amp-AgNps. The second weight loss corresponds to the decomposition of ampicillin in a temperature range of 218 to 565 °C that may be the result of covalent interaction of an amine group with silver nanoparticles indicating that higher energy is required for the desorption of ampicillin from silver nanoparticles. The loss value of nanoparticles is 54.2% and approximately 32% of silver was calculated using TGA in Amp-AgNps. The results suggest that the loss value of pure ampicillin is 86.4%, hence the efficient amount of ampicillin on silver nanoparticles is about 62.5%. Lastly the weight loss was observed at higher temperature region around 565 to 610 °C which is most likely due to the electrostatic interaction of the amine group. To validate that amine group is responsible for the synthesis of Amp-AgNps computational studies was done. Molecular dynamics (MD) simulation of ampicillin upon Ag^+^ (Amp-AgNps) binding was carried out to evaluate the stability of a system under exploited solvent condition. RMSD plot suggested that the binding of silver ion stabilized the ampicillin. It has been observed that Amp-AgNps structure underwent a deep transition of 0.641 nm to 3.93 nm and system got optimized at 2.7 nm (Fig. 4A). Ampicillin consists of 3 rings that is benzene ring, beta-lactam ring and thiazolidine ring. To study the interaction of ampicillin and silver particle, simulation at different time scale were done. The results clearly indicate that in aqueous state silver particle is interacting with the amine group of benzene ring of ampicillin (Fig. 4B). The structure obtained at different time frames were dumped which shows the interaction of sliver particle with benzene ring (Fig. 4C). Further, number of contacts that silver is making with these rings were calculated and plotted as a graph to show the contacts between silver and different rings of ampicillin (Fig. 4D). The graph clearly shows that sliver particle is making highest number of contacts with the benzene ring.

The antibacterial properties of Amp-AgNps were studied against sensitive and drug resistant gram positive and gram negative bacteria and the results were compared with purchased chemically synthesized silver nanoparticles (Ch-AgNps). Table 1 shows the MIC values of Amp-AgNps and Ch-AgNps with different bacterial strains in which MIC of ampicillin sensitive *E. coli* and *S. aureaus* was found to be 18.75 and 9.375 µg/ml whereas Ampicillin resistant *E. coli* and *S. aureus* shows 90% inhibition at 10 and 3 µg/ml. The susceptibility of Amp-AgNps was also determined against multidrug resistant bacteria *P. aeruginosa* and *K. pneumonia*, the MIC was found to be 20 µg/ml and 28.12 µg/ml respectively. The *P. aeruginosa* and *K. pneumonia* used were resistant to cephalosporins, aminoglycosides, carbapenems and ß-lactamase inhibitors. The MIC value of purchase (Sigma) silver nanoparticles was in the range of 280–720 µg/ml, thus linking an antibiotic to nanoparticles has significantly enhances the antibacterial properties due to synergistic effect. The enhanced bactericidal activity of antibiotic adsorbed silver nanoparticles against biorecycling microbes has also been reported by Khurana *et al*.^24^.

**Table 1:** MICs of test compounds against different bacterial strains.

Strain	MIC_90_ (µg/ml)		
	Amp-AgNps	Ch-AgNPs	Ampicillin
*E. coli*	18.75	≥320	≥12
*S. aureaus*	9.375	≥280	≥16
Ampicillin Resistant *E. coli*	10	≥320	≥256
Ampicillin Resistant *S. aureus*	3	≥300	≥125
*K. pneumonia* (MDR)	28.12	≥512	≥720
*P. aerigunosa* (MDR)	20	≥640	≥720

Further sensitivity of Amp-AgNps was evaluated on solid media by disk diffusion assay using luria agar. The results of disk diffusion assay are summarized in Table 2. Interestingly the ampicillin at a concentration of synthesis of silver nanoparticles (0.4 mM) and silver nitrate alone does not give any zone of inhibition.

**Table 2:** Disk diffusion assay of Amp-AgNps against different bacterial strains.

Concentration (µg)	Zone of inhibition (mm)					
	Sensitive *E. coli*	Sensitive *S. aureaus*	Ampicillin Resistant *E. coli*	Ampicillin Resistant *S. aureus*	*K. pneumonia* (MDR)	*P. aeruginosa* (MDR)
AgNp-5	7	8.5	15	13	7	8.5
AgNp-10	16	10	24	16	11	13
AgNp-20	19	13	26	23	15	16.5
AgNO_3_	—	—	—	—	—	—
Control	—	—	—	—	—	—
Ampicillin	10	8	—	—	—	—

Recently the emergence of resistance in gram negative bacteria *Escherichia coli* and *Pseudomonas aeruginosa* after repeated exposure to chemically synthesize silver nanoparticles has been reported^21^. This interesting study motivates us to give repeated exposure of Amp-AgNps to overcome bacterial resistance in sensitive and resistant bacterial strains. For this, all the bacteria were exposed to minimum inhibitory concentrations of Amp-AgNps and grown on luria agar plate, colonies were isolated and inoculated in fresh culture media. For second cycles the preceding inoculum were exposed to MIC concentration of Amp-AgNps. The same step is repeated for 15 consecutive cycles (Table 3). The results indicate that the MIC values of Amp-AgNps in all cycles (15 cycles) remain constant for all the tested strains and there is no emergence of resistant among these strains against Amp-AgNps after repeated usage. The results indicate the advantage of using ampicillin as precursor for synthesis of silver nanoparticles.

**Table 3:** Minimum inhibitory concentrations of Amp-AgNps for 15 successive cycles against different bacterial strains.

Strain	MIC_90_ (µg/ml)														
	1	2	3	4	5	6	7	8	9	10	11	12	13	14	15
*E. coli*	18.75	18.75	17.8	18.55	17.67	18.75	18.75	18.2	18	18.34	18.25	18	18.25	18.25	18
*S. aureaus*	9.375	9	9.25	8.95	9.23	8.75	9	9	9.25	9.25	9	9.3	9	9	9.25
Ampicillin Resistant *E. coli*	10	10	10	10.4	9.8	9.25	10	10.25	9.85	9.4	10	10.4	9.8	10	10
Ampicillin Resistant *S. aureus*	3	2.75	3	3.32	3	3.35	3	2.75	3.5	3.2	3.2	3	3	3.2	3
*K. pneumonia* (MDR)	28	28.12	28.12	28	28.2	28.2	28.45	28	28	28.12	28.12	28	28	28.12	28
*P. aerigunosa* (MDR)	20	20.4	20.67	19.43	20	20	20.89	20	20.4	19.25	19.43	20	19	20.4	20

**Figure 5:**
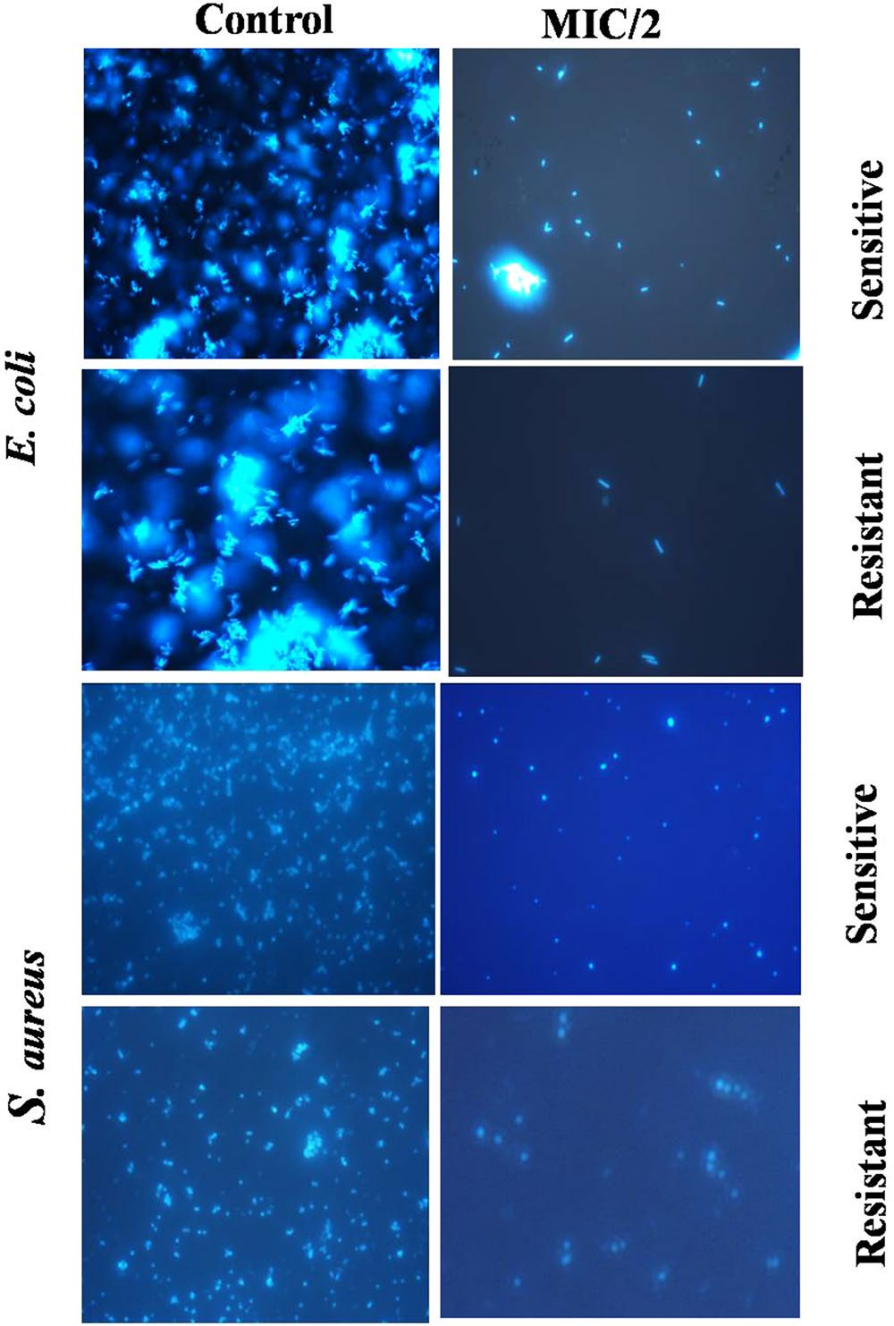
Fluorescence microscopy images of *E. coli* and *S. aureus* cells treated with and without Amp-AgNps at MIC/2 concentrations for 6 h, subsequently stained 30 min with DAPI.

**Figure 6:**
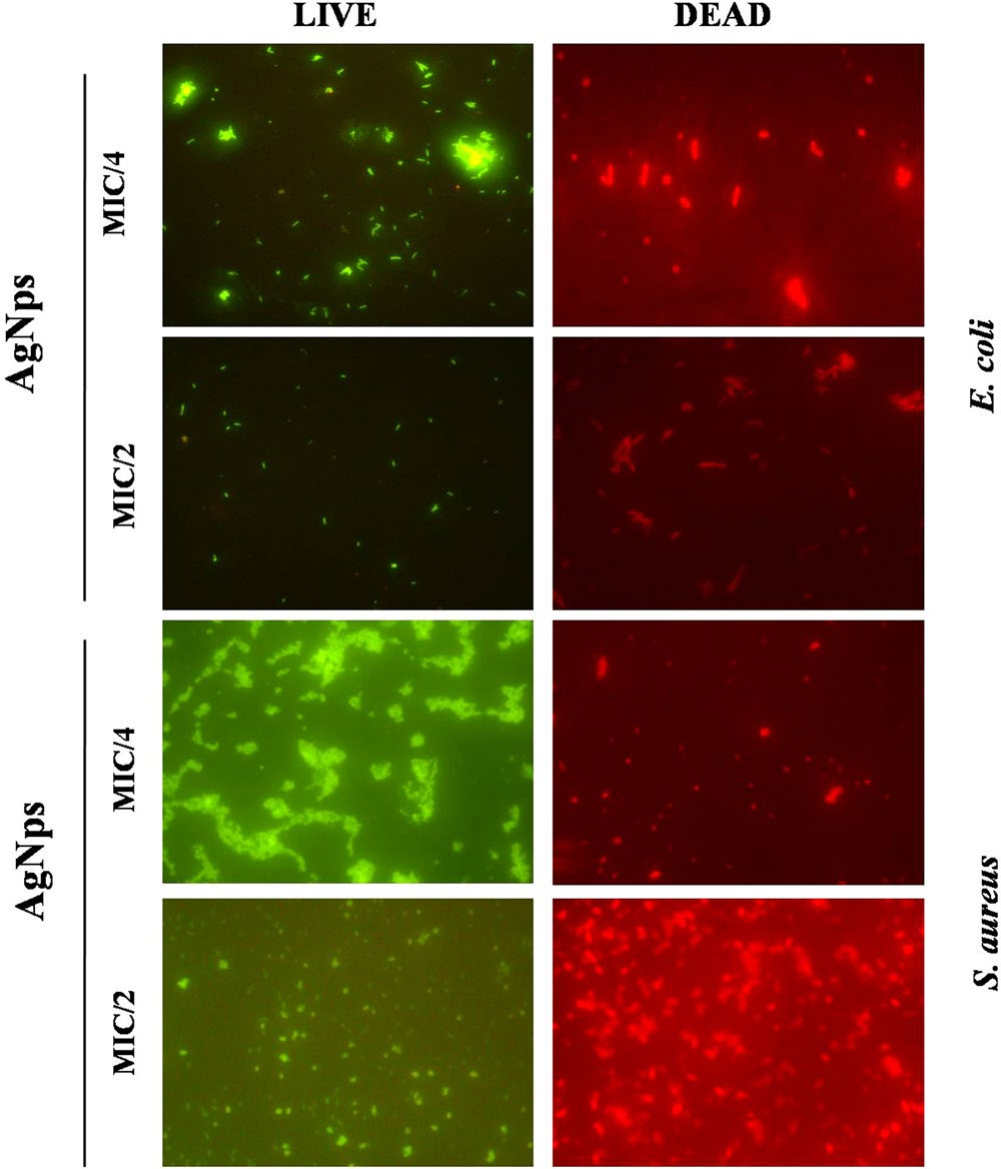
Fluorescence microscopy images of *E. coli* and *S. aureus* cells treated with Amp-AgNps at MIC/4 & MIC/2 concentrations for 6 h, subsequently stained for 30 min with SYTO9 (green) and PI (red) for antibacterial activity. Cells with green stand for live bacteria, while the red cells are representative of dead bacteria.

**Figure 7:**
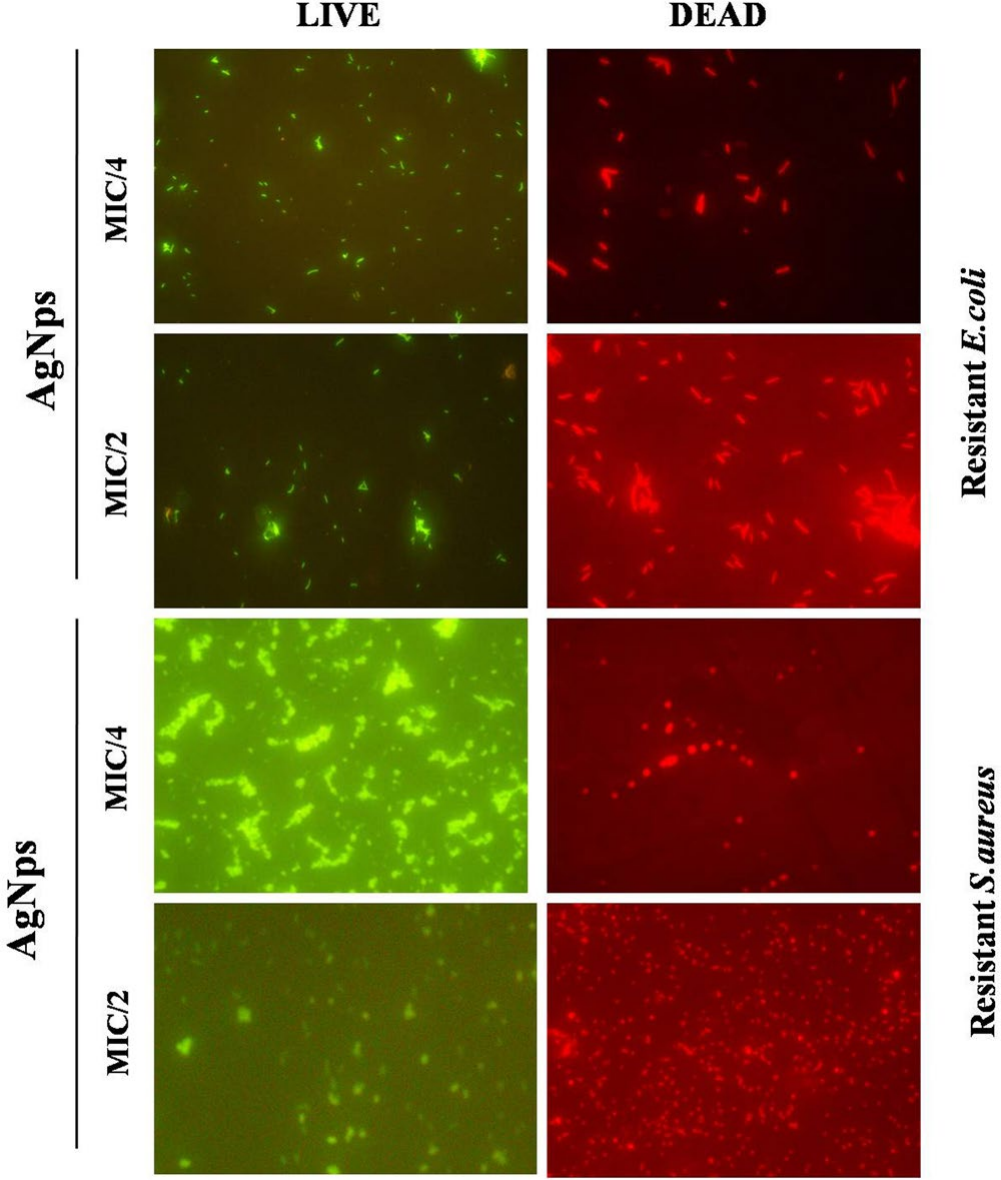
Fluorescence microscopy images of Resistant *E. coli* and *S. aureus* cells treated with Amp-AgNps at MIC/4 & MIC/2 concentrations for 6 h, subsequently stained 30 min with SYTO9 (green) and PI (red) for antibacterial activity. Cells with green stand for live bacteria, while the red cells are representative of dead bacteria.

To study the mechanism of action of Amp-AgNps the bacterial cells were stained with fluorescent dye DAPI which strongly binds to A = T rich regions in DNA. Figure 5 shows the microscopic images of ampicillin sensitive and resistant bacterial strains with and without Amp-AgNps. The blue cells are the viable bacterial cells which are stained by DAPI, at low concentrations of Amp-AgNps the number of viable cells are more as the concentration of Amp-AgNps increases the number of viable cells decreases. On the basis of above results it may be inferred that bacteria can uptake Amp-AgNps and disrupt the DNA leading to cell death. To study the live/dead bacteria another fluorescence assay was performed using propidium iodide and SYTO 9 dyes and the results are shown in Figs 6 and 7. The staining was performed at 488 nm laser excitation and 530 nm emission filters for SYTO 9 and at 561 nm laser excitation and 640 nm emission filters for propidium iodide. After the incubation of treated and untreated bacterial cells with propidium iodide and SYTO 9 respectively, the Amp-AgNps treated cells were stained intensely red which indicates the dead cells whereas untreated cells were fluorescing green which indicates the live cells. Moreover the results demonstrate that at low concentrations of Amp-AgNps (MIC/4) shows higher number of live bacteria and lesser number of dead bacteria whereas at higher concentrations of Amp-AgNps (MIC/2), the number of live cells decreases and dead cells increases. Hence these results confirmed the bactericidal action of silver nanoparticles which may be due to cell destruction and inhibition of cellular propagation.

**Figure 8:**
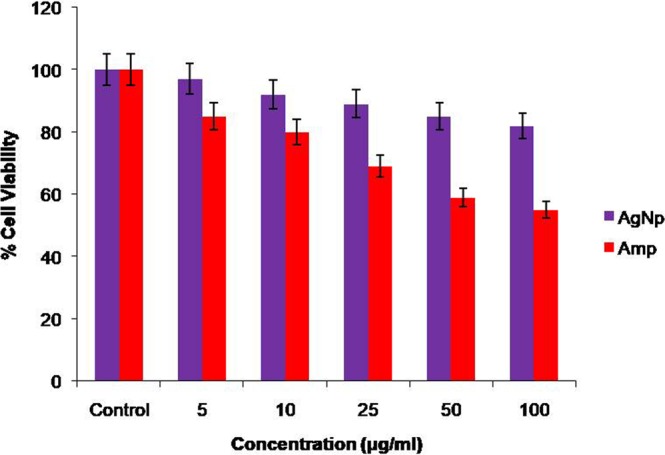
Dose-dependent effect of ampicillin and Amp-AgNps against HaCaT cell lines. The percent viable cells were calculated in comparison to untreated cells taken as 100%. Values were expressed as mean ± SD the experiment, performed in triplicate. Statistical significance was assessed by the Dunnet’s t-test: p < 0.05 was considered significant.

For antibacterial drugs and clinical applications, the cytotoxicity of Amp-AgNps against keratinocytes cell lines HaCaT was explored using MTT assay. The Amp-AgNps are found to be non toxic (Fig. 8) to mammalian cells with significant antibacterial activity against ampicillin resistant and multidrug resistant bacteria. This may be due to difference in the composition of cell membrane of bacteria and mammal. The bacterial membrane consists of peptidoglycan layer made up of sacchride units and majorly hydrolysis of bonds of sacchride units of peptidoglycan layer lead to cell death of bacteria. It is well established that ampicillin inhibits enzymes present in the bacterial cell walls, such as transpeptidases and carboxypeptidases which are mainly responsible for the synthesis of peptidoglycan layer of bacterial cell walls^25^. The literature clearly indicates that β lactam antibiotics resemble d-alanylalanine peptide fragment an enzyme substrate which facilitates binding of penicillin-binding proteins. These penicillin binding proteins are a group of enzymes found anchored in the cell membrane, which are involved in the cross-linking of the bacterial cell wall. The antibiotic irreversibly binds to the active site of these enzymes, disrupting cell wall synthesis. As the peptidoglycans are absent in human cells so antibiotics are not able to affect them^25^.

## Conclusions

A green approach is developed to synthesize silver nanoprticles using ampicillin. The β lactum ring of antibiotic does not participate in reduction as suggested by FTIR and computational MD simulation. Thus, the synthesized silver nanoparticles show potent antimicrobial activity as compared to chemically synthesized nanoparticles. Moreover they are effective against MDR. The repeated exposure of bacterial cultures with Amp-AgNps does not show any emergence of resistance. Thus these Amp-AgNps can be used as drugs against the resistant bacteria as these are non toxic as shown by MTT assay. Future studies should be carried out to modify silver nanoparticles with different kinds of drugs and such studies can be beneficial in combating the emerging threat of resistance among the bacteria.

## Materials and Methods

### Materials

Silver nanoparticles (10 nm) and Silver nitrate were purchased from Sigma Aldrich. MTT (3-(4,5-dimethyl-2-yl)-2,5-diphenyl tetrazolium bromide), Dulbecco’s modified Eagle’s medium (DMEM), Trypsin, EDTA mixture and antibiotics were procured from HiMedia. Fetal Bovine Serum (FBS) was obtained from Gibco (Grand Island, NY).

### Methods

#### Synthesis and purification of silver nanoparticles

Optimization: The synthesis of silver nanoparticles was optimized by varying the different concentration of silver nitrate (2–10 mM), Ampicillin (2–15 mM), temperature (40–70 °C) and time by scanning the samples in the wavelength range of 300–700 nm using double beam UV Vis spectrophotometer.

Synthesis: The synthesis of silver nanoparticles was done by incubating 20 ml of ampicillin (10 mM dissolved in distilled water) and 10 ml of silver nitrate (10 mM dissolved in distilled water) solutions and final volume was made to 500 mL by adding distilled water and kept at 60 °C on water bath for 3.5 hr. Silver nanoparticles formed were separated by a simple process of centrifugation at 16500 g, 4 °C for 20 mins as described earlier^14^.

Stability: The colloidal solution of silver nanoparticles was kept at room temperature for three months and surface Plasmon resonance was recorded using UV Vis spectrophotometer.

#### Characterization of silver nanoparticles

UV-Vis Spectroscopy: The optical properties of synthesized silver nanoparticles were measured by Mecasys Optizen 3220UV spectrophotometer in the wavelength range from 300 to 700 nm 10 dm path length quartz cell and 2 nm slit width with the resolution of 5 nm.

#### Dynamic light scattering

DLS measurements for determining the mean diameter, Zeta potential and polydispersity index of the silver nanoparticles were carried out using the spectroscatterer RiNA, GmbH class3B. The dried powder was dispersed in distilled water to produce a suitable scattering intensity and values were obtained at an angle of 90° in 10 mm diameter cells at 20 °C for 10 cycles.

TEM measurements: TEM was performed on a JEOL model JEM-2000FX instrument operated at an accelerating voltage of 200 kV. TEM samples were prepared by placing a drop of sonicated powdered sample, in absolute ethanol for about 15 min on ultrasonicator (UP-500 Ultrasonic Processor), on a carbon coated copper grid and dried in air for 1 h.

X-ray diffraction (XRD): XRD pattern was recorded by X’PertPro X-ray diffractometer (PANalytical B.V.) by using Cu- Kα radiation and operating X-ray tube at 45 kV and 35 mA.

Fourier transform infrared (FTIR): Spectrum of ampicillin and purified silver nanoparticles were done on a Perkin Elmer FTIR spectroscopy using KBr pellets. To obtain good signal to noise ratio, 32 scans of silver nanoparticles and ampicillin were taken in the range 400–4000 cm^−1^ and the resolution was kept as 4.0 cm^−1^.

Thermogravimetric analysis (TGA): TGA analysis of ampicillin mediated silver nanoparticles and ampicillin powder was performed using a Q-500 TGA from TA instrument (New Castle, USA) over a temperature range of 30–200 °C at a heating rate of 10 °C/min in the presence of N_2_ gas.

#### Molecular dynamics simulation

Molecular dynamics simulations were performed using the GROMACS version 5.1 software^26^ with the standard CHARMM36m force field^27^. The ampicillin was soaked in a cubic box of water molecules with a dimension of 5 Å i.e. setting the box edge 5 Å from the molecule perimeters, using the editconf module for making boundary conditions and solvate for solvation. For the solvation of proteins, the spc216 template was used. The system was then minimized using the 5000 steps of steepest descent at 310 K during their equilibration period (100 ps) at a constant volume under periodic boundary conditions. Equilibration was performed in two phases: NVT ensemble (constant number of particles, volume, and temperature at 100 ps) and NPT ensemble (constant number of particles, pressure, and temperature at 100 ps). The resulting trajectories were analyzed, using RMSD by the utilities provided by GROMACS. For ampicillin topology, SwissParam server was used^28^ that provides topologies and parameters for small organic molecules, compatible with the CHARMM all atoms force field, for use with the CHARMM or GROMACS Softwares^28,29^.

#### Microorganism and culture conditions

Bacterial strains were purchased from Microbial Type Culture Collection (MTCC), Institute of Microbial Technology (Chandigarh, India). *E. coli*, *S. aureaus*, ampicillin resistant *E. coli*, *S. aureaus*, multi drug resistant *Pseudomonas aeruginosa* and *Klebsiella pneumonia* were cultured in Luria broth. Cells were maintained at 37 °C.

#### Minimum inhibitory concentration (MIC)

The MIC was evaluated by 96-well micro dilution method according to published protocols^30^.

#### The agar diffusion test or bauer-kirby test

The antibacterial efficacy of Amp-AgNps was evaluated by agar diffusion method as described earlier^14^.

#### Culture of bacteria in presence of Amp-AgNps; to check resistance

The bacterial culture at a concentration of 10^6^ CFU ml^−1^ (where CFU is colony-forming unit) were incubated at sub inhibitory concentration of Amp-AgNps and incubated at 37 °C for 24 hours, MIC was determined. After 24 hours the 2 µl of treated bacterial suspension were streak on luria agar plates and incubated at 37 °C for 24 hours. The bacterial colony developed were isolated and inoculated into fresh luria broth, incubated at 37 °C for 24 hours. For the next cycle bacterial culture prepared were exposed to MIC concentrations of Amp-AgNps at a concentration of 10^6^ CFU ml^−1^ and MIC were determined again. The same process was repeated upto 15 successive cycles and MIC was calculated in each cycle. The experiments were repeated thrice.

#### Fluorescence microscopy

The bacterial cells (*E. coli* and *S. aureus*) were harvested by centrifugation at 3000 rpm, 5 min, washed twice with phosphate buffer saline (PBS, pH 7.5, 0.1 M) and O.D. was adjusted to 1.25 at 595 nm. The cells were incubated at MIC/4 and MIC/2 concentrations of Amp-AgNps for 5 h at 37 °C, 180 rpm. A control was run in which bacterial cells without Amp-AgNps were studied by keeping all the parameters same. The bacterial suspensions were counter stained with DAPI, SYTO9 and propidium iodide for 30 min in the dark followed by washing with PBS twice. The slides were prepared by smearing 2 μL of different kinds of bacterial suspensions on microscopic slide and samples were observed at Nikon Eclipse 80i Advance Research Fluorescence microscope.

#### Cytotoxicity assay

MTT assay was used to study the cytotoxic effect of Amp-AgNps against HaCaT (Keratinocytes) cells. HaCaT cells were seeded in 96-well plates at a density of 10^4^ cells per well in 0.2 ml of DMEM: Ham’s F12 (1:1 v/v) with 10% FBS and 1% antibiotics and was cultured at 5% CO_2_ and 37 °C for 24 h. Growth medium in the wells was replaced after 24 h with medium containing Amp-AgNps (0.156–100 μg/ml) and incubated for 24 h. The medium was removed thereafter and replaced with 100 μl of medium containing 3(4,5-dimethyl-thiazol-2-yl)-2,5-diphenyltetrazolium bromide (MTT) and incubated for 4 h. The unreduced MTT was taken out, and 200 μl of DMSO was added to each well to dissolve the MTT formazan crystals. The content was mixed properly, and absorbance was measured at 595 nm in a microplate reader (iMark MicroplateAbsorbance Reader, BioRad, USA)^31^.

Percentage cytotoxicity was calculated as a fraction of control (untreated) and the cytotoxicity was expressed in IC_50_.

### Statistical analysis

Analysis of variance (ANOVA) was performed followed by Dunnett’s test and values were represented as means of three replicates (n = 3) ± SD. The significance level was maintained as p- value < 0.05.
